# Supplementary material for: Blue-wavelength light therapy for post-traumatic brain injury sleepiness, sleep disturbance, depression, and fatigue: A systematic review and network meta-analysis
Source: PLoS One. 2021 Feb 4;16(2):e0246172. doi: 10.1371/journal.pone.0246172 (PMC7861530; doi:10.1371/journal.pone.0246172)
Supplement: S1 Text — (PDF) [file pone.0246172.s004.pdf]

S1 Text. Search strategies (July 4, 2020).

Pubmed: 394
(light OR phototherapy) AND (((head OR brain) AND (trauma* OR injur*)) OR concussion) AND random*

Scopus: 572
TITLE-ABS-KEY ((light OR phototherapy) AND (((head OR brain) AND (trauma* OR injur*)) OR concussion) AND random*)

Web of science: 326
You searched for: TOPIC: ((light OR phototherapy) AND (((head OR brain) AND (trauma* OR injur*)) OR concussion) AND random*)
Timespan: All years. Indexes: SCI-EXPANDED, SSCI, A&HCI, ESCI.

Cochrane CENTRAL: 179
(light OR phototherapy) AND (((head OR brain) AND (trauma* OR injur*)) OR concussion) AND random* in Title Abstract Keyword

Academic Search Complete: 160
(light OR phototherapy) AND (((head OR brain) AND (trauma* OR injur*)) OR concussion) AND random*

CINAHL Complete: 66
(light OR phototherapy) AND (((head OR brain) AND (trauma* OR injur*)) OR concussion) AND random*
