# Supplementary material for: Blue-wavelength light therapy for post-traumatic brain injury sleepiness, sleep disturbance, depression, and fatigue: A systematic review and network meta-analysis
Source: PLoS One. 2021 Feb 4;16(2):e0246172. doi: 10.1371/journal.pone.0246172 (PMC7861530; doi:10.1371/journal.pone.0246172)
Supplement: S1 Fig — (PDF) [file pone.0246172.s005.pdf]

S1 Fig. Risks of bias assessed using the RoB (Risk of Bias) 2 tool for randomized trials[1,2]

(A) Within individual trials

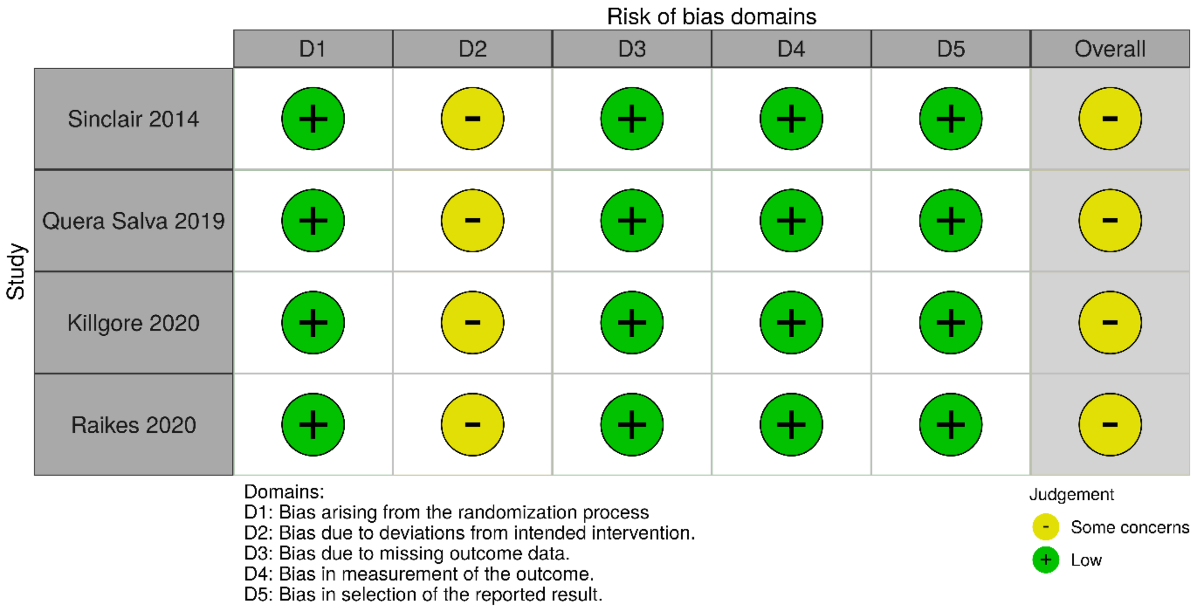

(B) Across included trials

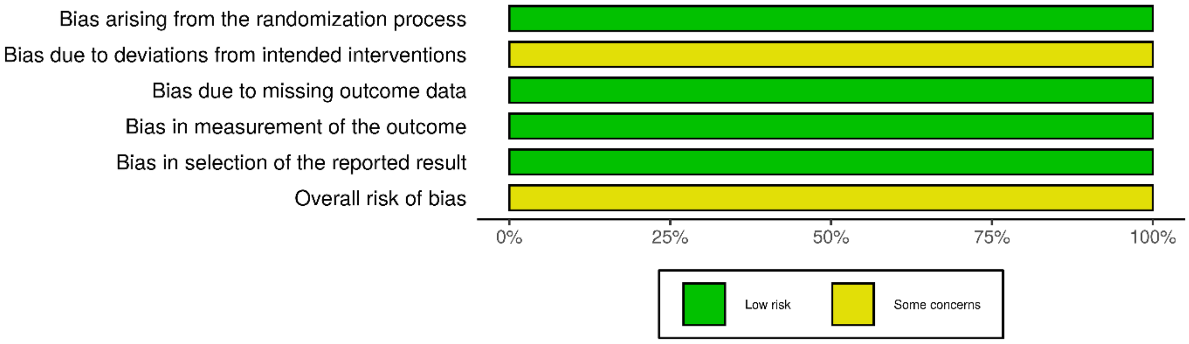

1. Higgins J, Thomas J, Chandler J, et al. *Cochrane Handbook for Systematic Reviews of Interventions*. 2nd Edition. Chichester (UK): John Wiley & Sons, 2019.

2. McGuinness LA, Higgins JPT. Risk-of-bias VISualization (robvis): An R package and Shiny web app for visualizing risk-of-bias assessments. *Res Synth Methods*. Epub ahead of print 26 April 2020. DOI: 10.1002/jrsm.1411.
