# Supplementary material for: Blue-wavelength light therapy for post-traumatic brain injury sleepiness, sleep disturbance, depression, and fatigue: A systematic review and network meta-analysis
Source: PLoS One. 2021 Feb 4;16(2):e0246172. doi: 10.1371/journal.pone.0246172 (PMC7861530; doi:10.1371/journal.pone.0246172)

S2 Fig. Funnel plots to visualize the publication bias of five outcomes: (A) for sleepiness; (B) for sleep disturbance; (C) for depression; (D) for fatigue; and (E) for dropout rates.

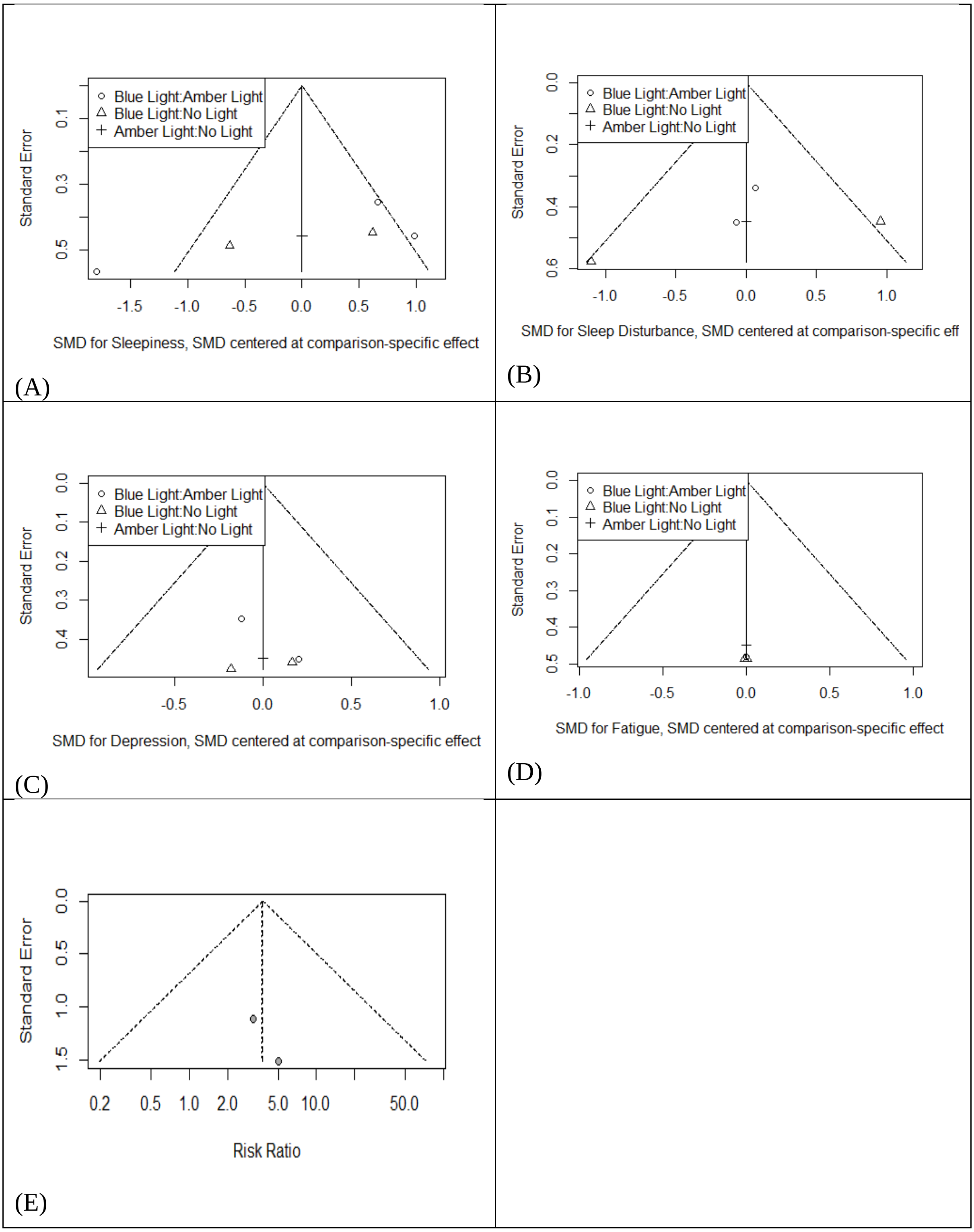

Supplement: S2 Fig — (PDF) [file pone.0246172.s006.pdf]
